# Supplementary material for: Market-based instruments to fund nature-based solutions for flood risk management can disproportionately benefit affluent areas
Source: Commun Earth Environ. 2025 Aug 28;6(1):714. doi: 10.1038/s43247-025-02706-2 (PMC12394074; doi:10.1038/s43247-025-02706-2)
Supplement: Supplementary file 2 — Supplementary Information [file 43247_2025_2706_MOESM2_ESM.pdf]

Supplementary Table 1: This table provides a detailed description of each dataset, with access made available upon request.

Name of Dataset	*Format*	*Scale*	*Description/Purpose*	*Year Published*
NFM_Assets_v2.4_2021	.shp	Point (Lat, Long)	4,428 individual NFM assets created between 2017 – 2021. Used to identify discrepancies between projects and individual assets.	2021
DEFRA_15m_NFM_Pilot_Projects_2021	.shp	Point (Lat, Long)	86 Individual NFM projects cleaned down to 34 community projects for 2017 – 2021 funding. Used to identify the locations that received NFM funding from DEFRA grants.	2021
NFM_25m_NFM_Projects_2023	.shp	Point (Lat, Long)	41 Individual Projects created in ArcGIS Pro for 2023+ funded NFM projects. Identified manually, and used to compare how funding inequalities may have changed between 2017 and 2023.	N/A
LSOA_IMD_2019_(OSGB1936)	.shp	Polygon (LSOA)	33,755 Lower Super Output Area polygons linked to the Index of Multiple Deprivation 2019 values by rank and decile. Used to spatially join the location of NFM features to deprivation scores.	2019
RUC_LSOA_2011	.csv	LSOA (Coded)	33,755 Lower Super Output Area cell values linked to the Rural Urban Classification 2011 by code and name.	2011

			Used to classify NFM features as either rural or urban to identify different dynamics between classes.	
MSOA_DEC_2021	.shp	Polygon (MSOA)	7,264 Middle Super Output Area polygons unlinked to any data. This is simply for joining the age by single year information.	2021
Age_by_single_year _2021	.xlsx	MSOA (Coded)	7,264 Middle Super Output Area cell values for Age in 2021. This data was processed to create an average age per MSOA, and then joined to the MSOA_2021 polygons.	
£25m Application Pack	.zip folder	N/A	A collection of documentation on the screening and application process for the 2023 NFM programme. Used to identify areas of potential inequality in the application process.	N/A
FOI-NR366962	.xlsx	Point (Grid Referenced)	An Excel document containing the Non-Funded Applications for both the 2017 and 2023 NFM funding schemes. Used to identify the demographics of projects that were not selected for funding compared to where was funded.	N/A
Project Proposal Application Form	.xlsx	N/A	An example of the application form for the 2023 funding which	N/A

			can be referenced to understand the process of application to the grant.	
HoC GE2019 results by constituency	.xlsx	Constituency	7,638 Electoral Wards showing the results of the winning seat for each ward in 2019's General Election, UK.	2017
HoC GE2017 results by constituency	.xlsx	Constituency	7,638 Electoral Wards showing the results of the winning seat for each ward in 2017's General Election, UK.	2017
England_and_Wales_LSOA_flood_and_social_vulnerability_data	.xlsx	LSOA (Coded)	33,755 Lower Super Output Area cell values showing if the LSOA is affected by <10%, 10%< x <50%, or <50% flooding. Used to identify whether the LSOA selected for NFM funding has an overall high-risk of flooding for residents.	2023
